# The Yeast Shu Complex Utilizes Homologous Recombination Machinery for Error-free Lesion Bypass via Physical Interaction with a Rad51 Paralogue

**DOI:** 10.1371/journal.pone.0081371

**Published:** 2013-12-05

**Authors:** Xin Xu, Lindsay Ball, Wangyang Chen, Xuelei Tian, Amanda Lambrecht, Michelle Hanna, Wei Xiao

**Affiliations:** 1 College of Life Sciences, Capital Normal University, Beijing, China; 2 Department of Microbiology and Immunology, University of Saskatchewan, Saskatoon, Canada; King Faisal Specialist Hospital & Research center, Saudi Arabia

## Abstract

DNA-damage tolerance (DDT) is defined as a mechanism by which eukaryotic cells resume DNA synthesis to fill the single-stranded DNA gaps left by replication-blocking lesions. Eukaryotic cells employ two different means of DDT, namely translesion DNA synthesis (TLS) and template switching, both of which are coordinately regulated through sequential ubiquitination of PCNA at the K164 residue. In the budding yeast *Saccharomyces cerevisiae,* the same PCNA-K164 residue can also be sumoylated, which recruits the Srs2 helicase to prevent undesired homologous recombination (HR). While the mediation of TLS by PCNA monoubiquitination has been extensively characterized, the method by which K63-linked PCNA polyubiquitination leads to template switching remains unclear. We recently identified a yeast heterotetrameric Shu complex that couples error-free DDT to HR as a critical step of template switching. Here we report that the Csm2 subunit of Shu physically interacts with Rad55, an accessory protein involved in HR. Rad55 and Rad57 are Rad51 paralogues and form a heterodimer to promote Rad51-ssDNA filament formation by antagonizing Srs2 activity. Although Rad55-Rad57 and Shu function in the same pathway and both act to inhibit Srs2 activity, Shu appears to be dedicated to error-free DDT while the Rad55-Rad57 complex is also involved in double-strand break repair. This study reveals the detailed steps of error-free lesion bypass and also brings to light an intrinsic interplay between error-free DDT and Srs2-mediated inhibition of HR.

## Introduction

It is vital for living organisms to protect their genomic integrity from a variety of endogenous and exogenous DNA damage. In *Saccharomyces cerevisiae*, Rad6 and Rad18 form a stable E2–E3 complex considered to initiate a pathway traditionally named DNA post-replication repair (PRR). As genes falling into the *RAD6* epistasis group function to bypass replication blocks instead of the actual removal of the lesions encountered, this pathway has been renamed DNA-damage tolerance (DDT) [1]. DDT is defined as a survival mechanism to deal with stalled replication forks in the presence of DNA damage, and has been revealed to consist of two parallel branches, error-free and error-prone lesion bypass [2], [3], [4].

Activation of DDT and the regulation of either error-free or error-prone lesion bypass is achieved via sequential modifications of proliferating cell nuclear antigen (PCNA) [5]. In the budding yeast, PCNA is encoded by the *POL30* gene and forms a homotrimer to anchor on the DNA like a sliding clamp [6]. When PCNA encounters a replication block, its K164 residue is monoubiquitinated by the Rad6-Rad18 complex [5], which facilitates error-prone translesion synthesis (TLS) represented by Polη (Rad30), Rev1 and Polζ (consisting of Rev3 and Rev7 subunits) [7], [8], [9]. When the monoubiquitinated PCNA is further polyubiquitinated by the Rad5-Ubc13-Mms2 E2–E3 complex via the formation of a K63-linked polyUb chain, it is thought to promote error-free lesion bypass [5]. Unlike TLS, little is known about how the error-free DDT pathway operates [10]. It has been well accepted that the error-free DDT branch can restore the original information via homologous recombination (HR) by using an intact sister chromatid as a template [4], [11], [12], but the detailed molecular cascade is largely missing.

A key protein for homologous recombination (HR) in eukaryotes including budding yeast is Rad51, a RecA-like protein [13], [14]. In order for strand invasion to take place and HR to proceed, Rad51 must replace replication protein A (RPA) with the help of Rad52 from single-stranded DNA (ssDNA) overhangs and form a nucleoprotein filament [15], [16], [17]. In the absence of replication-blocking lesions, PCNA can be covalently modified at the K164 residue by a small ubiquitin-like modifier (SUMO), which recruits Srs2 [18], [19], a putative anti-recombinase, and suppresses HR by releasing Rad51 from ssDNA [20], [21], [22].

Rad55 and Rad57 are two Rad51 paralogues and function as a heterodimer to promote DNA strand exchange by Rad51 recombinase [23] and protect the Rad51 filament from the invasion of Srs2 [24]. Csm2 and Psy3 are also regarded as Rad51 paralogues and form a heterodimer as well; they share little sequence homology with Rad51 but have a similar structure [25], [26]. Interestingly, components of the Shu complex have been reported to have an Srs2-inhibiting function [27]. Like other HR proteins, mutations in the Shu complex can suppress the *top3Δ* slow growth and DNA damage sensitivity phenotypes [28], [29]. It has been well established that the Shu complex plays an important role in recruiting HR proteins into the error-free DDT [30]. However, how this is achieved remains unclear. Here we report that the Shu complex fulfills its roles by recruiting the Rad55-Rad57 complex and the HR machinery to the DDT sites to complete error-free DDT. The similarities and differences between these two Rad51 paralogues are explored in this study.

## Results

### rad55 and rad57 are Epistatic to Shu

**Figure 1:**
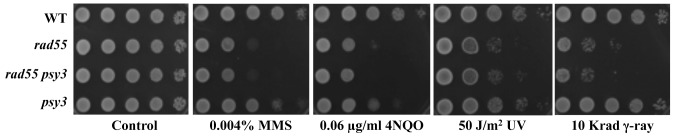
*rad55* is epistasic to *psy3* with respect to different DNA damage treatments. Cells grown overnight were spotted on YPD, YPD + MMS, or YPD + 4NQO at indicated concentrations by a tenfold serial dilution assay. For UV and γ-ray irradiations, spotted plates were exposed to the radiation at indicated doses. The plates were then incubated at 30°C for 2 days before photography. For each type of DNA damage, several doses were applied and only one is presented in the figure.

We have previously demonstrated that the Shu complex is involved in error-free DDT and perhaps functions at an early stage [30]. Since at least two subunits of the Shu complex (Csm2 and Psy3) are thought to be Rad51 paralogues and the Rad55–Rad57 complex is also a heterodimer of Rad51 paralogues, we hypothesized that these two complexes may confer redundant functions in facilitating HR. This hypothesis predicts that *shu* and *rad55/rad57* mutations are synergistic in response to DNA damage. In order to determine the genetic interaction between *shu* and *rad55/rad57*, we examined the phenotypes of *psy3* and *rad55/rad57* single null mutants and their corresponding double mutants with respect to killing by the X-ray and γ-ray mimetic agent methyl methanesulfonate (MMS). We used *PSY3* to represent *SHU* because it encodes a core protein of the complex that interacts with all three other components (Shu1, Shu2 and Csm2). Indeed, mutating all four genes does not further enhance sensitivity to DNA damage [29], [31]. The *rad55/rad57* single mutants are more sensitive to MMS than *psy3*; surprisingly, the *rad55/rad57 psy3* double mutants have the same sensitivity to MMS as the *rad55/rad57* single mutants (Figure 1 and Figure S1n in File S1), indicating that the two genes, and by extension the Shu and Rad55–Rad57 complexes, function in the same pathway. To further this observation, we examined the genetic relationship between the two complexes in response to a variety of representative DNA-damaging agents. The *psy3* mutant is slightly more sensitive to UV and 4-nitroquinoline oxide (4NQO) than the wild-type cells, but less sensitive than the *rad55* mutant (Figure 1). In contrast to *rad55*, the *psy3* mutant does not display noticeable sensitivity to γ-ray, suggesting that the Shu complex is not directly involved in repairing double-strand breaks (DSBs). In all cases, the *rad55 psy3* double mutant is no more sensitive than the *rad55* single mutant, suggesting that *rad55* is epistatic to *psy3*, or the two complexes function in the same pathway in a lesion-independent manner. Hence, we infer from the above observations that both complexes are required for bypassing replication-blocking lesions.

### Both the Shu and Rad55–Rad57 Complexes Function in the Same Error-free DNA-damage Tolerance Pathway

Genes involved in DDT are classified into two categories based on the phenotypes of their mutants. Mutants of genes in the first category show slight sensitivity to UV irradiation and other types of DNA damage, but their response to spontaneous and induced mutagenesis is compromised [32], [33]. In contrast, mutants in the second category are mildly sensitive to DNA damage with an associated increase in spontaneous and induced mutagenesis [34], [35], [36]. It becomes clear that the first category genes belong to the error-prone TLS branch, while the second category genes function in error-free DDT [3], [4].

**Figure 2:**
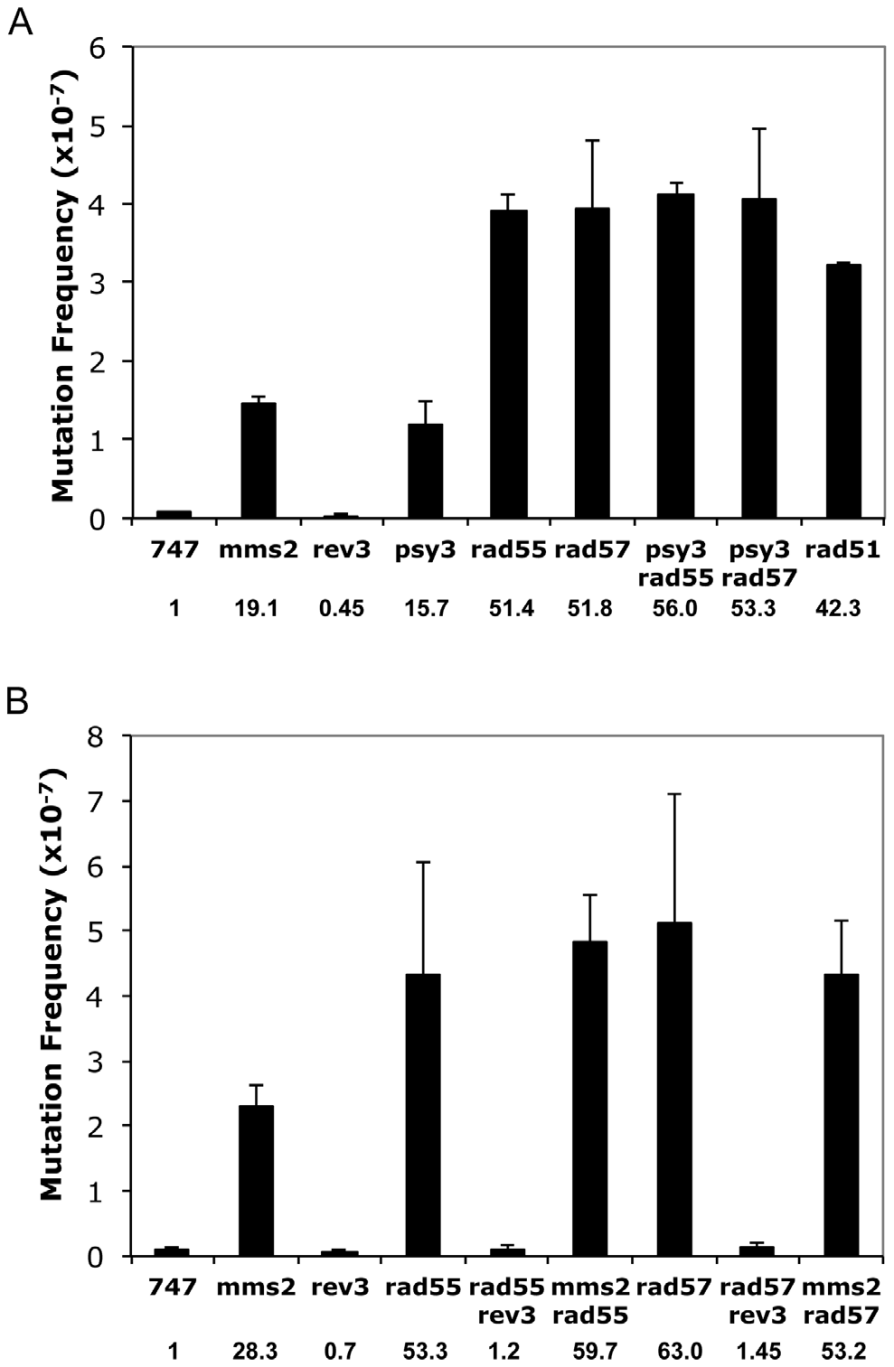
Deletion of *RAD55* or *RAD57* causes a massive increase in spontaneous mutagenesis in a TLS-dependent manner. The spontaneous mutation rates are determined by a *trp1-289* reversion assay in the DBY747 strain background. (A) *rad55/rad57* is epistatic to *psy3* with respect to spontaneous mutagenesis. *mms2* and *rad51* mutants are included as references. (B) *rad55/57* is epistatic to *mms2* with respect to spontaneous mutagenesis and the increased mutations are due to functional *REV3*. The presented data were from an average of at least three independent experiments with standard deviations. Relative levels of mutation rates are given in the bottom of each graph, expressed as a multiple of the wild-type level.

In a *trp1-289* reversion assay, *psy3* shows a dramatically increased mutagenesis rate comparable to the *mms2* mutant. Surprisingly, the spontaneous mutation rates in *rad55* and *rad57* mutants are even higher than those in *psy3* and *mms2* (Figure 2A). More importantly, no additional increase is observed when *rad55/rad57* mutation is combined with either *psy3* (Figure 2A) or *mms2* (Figure 2B), indicating that these genes work in the same pathway to limit spontaneous mutagenesis. Like *mms2* [35] and *psy3* [30], the increased mutation rates in *rad55/rad57* mutants are completely dependent on functional TLS, since once the *REV3* gene is deleted, the increased mutagenesis is completely abolished (Figure 2B). Hence, the Rad55–Rad57 complex acts in an error-free lesion bypass pathway whose inactivation channels lesions to the error-prone TLS pathway.

**Figure 3:**
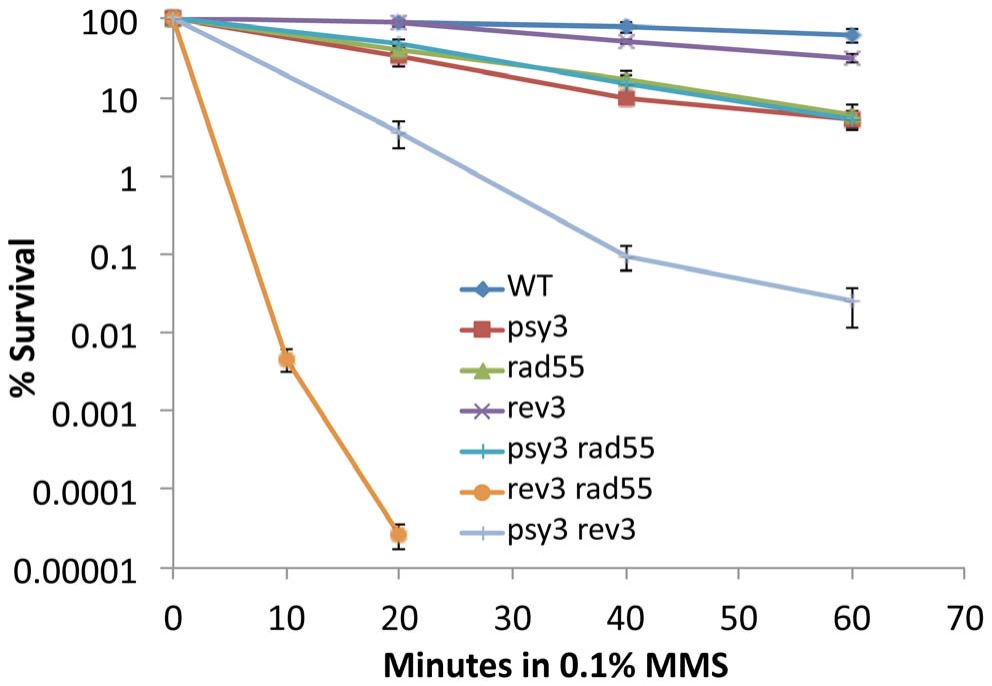
*rad55* is epistatic to *psy3* and synergistic to *rev3* with respect to MMS-induced killing. A time-course liquid-killing experiment was performed in the presence of 0.1% MMS as described in Materials and Methods. The results are the average of four independent experiments with standard deviations.

A characteristic phenotype of error-free lesion bypass mutation is its synergistic interaction with the TLS pathway mutation [37]. To further examine whether *RAD55* and *RAD57* genes belong to the error-free DDT pathway, we performed a liquid killing experiment. As shown in Figure 3, the *psy3* and *rad55* mutants have similar levels of sensitivity to killing by MMS and the double mutant is as sensitive as the single mutant, suggesting that they function in the same pathway. The *rev3* single mutant displays very moderate sensitivity; however, the *psy3 rev3* double mutant is much more sensitive to killing by MMS than either of the single mutants, and the effect is synergistic. Strikingly, the *rad55 rev3* double mutant is even more sensitive to MMS than the *psy3 rev3* double mutant, and the synergism is nearly 10^6^-fold more than if the effect were simply additive. Similarly, *rev3* is also synergistic with other HR pathway gene mutations known to carry out their functions downstream in the error-free branch of DDT (Figure S2 in File S1). Taken together, we conclude that like the Shu complex, the Rad55–Rad57 complex is also a component of error-free DDT and the two complexes function in the same pathway.

### Physical Interaction between Shu and Rad55–Rad57 Complexes via Csm2 and Rad55

**Figure 4:**
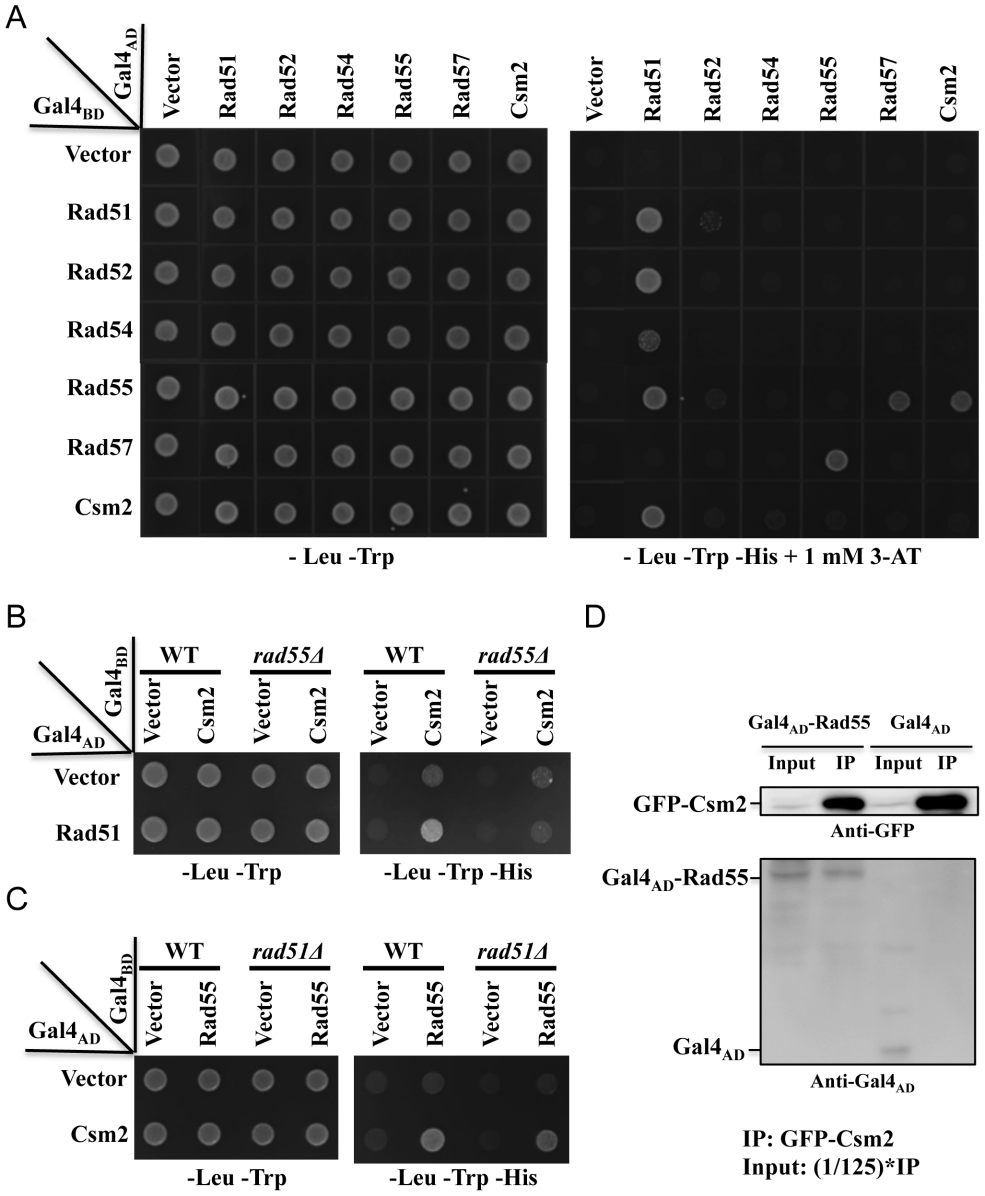
Physical interaction between Csm2 and Rad55. (A–C) Yeast two-hybrid analysis. PJ69-4a transformants carrying one Gal4_AD_ (from pGAD424) and one Gal4_BD_ (from pGBT9) derivative were replicated onto SD plates lacking certain amino acids as indicated and incubated for 3 days before being photographed. The same results are obtained from a reverse orientation assay. For each transformant, at least 4 independent colonies were taken for the functional assay and results shown in this figure contain one representative colony. (A) Physical interaction between Shu and HR proteins. (B) Effects of *rad55* mutation on the Csm2-Rad51 interaction. (C) Effects of *rad51* mutation on the Csm2-Rad55 interaction. (D) Coimmunoprecipitation to detect Csm2-Rad55 interaction. Total cell extracts from WXY3515 cells bearing plasmids pGAD-Rad55 or pGAD424 were subject to immunoprecipitation by GFP-Trap-A beads, and the products were analyzed by western blot using an anti-Gal4_AD_ antibody. Relative amount of samples is indicated in the figure legend.

Error-free DDT utilizes HR to restore the original information by employing the newly synthesized undamaged sister chromatid as the template, but how the HR machinery is recruited to the stalled replication fork remains unclear. Since Shu and Rad55–Rad57 act in the same pathway and the Rad55–Rad57 complex is considered part of the recombinosome [38], [39], we performed a systematic yeast two-hybrid assay between the Shu complex and HR proteins, and found that only Csm2 has a physical interaction with Rad55 as well as Rad51 (Figure 4A). Other Shu subunits do not interact with HR proteins (data not shown). From the matrix, we also notice that, as previously reported [31], [39], [40], [41], Rad51 can interact with all other HR proteins examined except Rad57 in one orientation, and Rad55 and Rad57 interact in both orientations consistent with a previous report that they function as a heterodimer [23]. In order to distinguish whether the interactions of Csm2-Rad55 and Csm2-Rad51 are dependent on each other, we carried out the yeast two-hybrid assay in the *rad55* or *rad51* null mutant background and found that the interaction between Csm2 and Rad51 depends on Rad55, since when *RAD55* is deleted, no Csm2-Rad51 interaction can be detected (Figure 4B). In contrast, deletion of *RAD51* does not appear to interfere with the Csm2-Rad55 interaction in the Y2H assay (Figure 4C). The above observations agree with a recent report [42]. We noticed a slightly reduced growth of *rad51* cells harboring *CSM2* and *RAD55* genes cloned into the Y2H vectors compared with those of wild-type cells. It was unlikely due to variations in replication, since all independent colonies showed similar effects. One possible explanation is that the Csm2-Rad55 interaction is partially dependent on Rad51. To further confirm the physical interaction between Csm2 and Rad55, we performed an *in vivo* coimmunoprecipitation experiment (Figure 4D), in which chromosomally integrated and GFP-tagged Csm2 is able to bind to Gal4_AD_-tagged Rad55 (lanes 1 and 2), while in the same experiment, GFP-Csm2 is unable to coimmunoprecipitate Gal4_AD_ alone (lanes 3 and 4). Hence, this interaction appears to be Csm2-Rad55 specific.

### *srs2* Suppresses *rad55* Sensitivity to Ionizing Radiation but not Other Types of DNA Damage

In addition to being ubiquitinated, PCNA can also be modified by a small ubiquitin-related modifier (SUMO) at K164, which provides a signal to recruit Srs2 to the replication fork to prevent the formation of the Rad51 protein filament on the ssDNA [18], [19]. *SRS2* was named because its mutant can suppress the sensitivity of the *rad6* mutant to UV radiation [43]. Indeed, *srs2* null mutation causes a hyper-recombination effect when exposed to UV radiation, and the suppression of *rad6* UV sensitivity is dependent on functional HR [20], [44], [45], [46]. Both Rad55–Rad57 and Shu complexes have been reported to be competitive and antagonistic with Srs2 [24], [27]. The Shu complex physically interacts with Srs2 via Shu2 [40], and this interaction is also reported in *Schizosaccharomyces pombe* [47].

**Figure 5:**
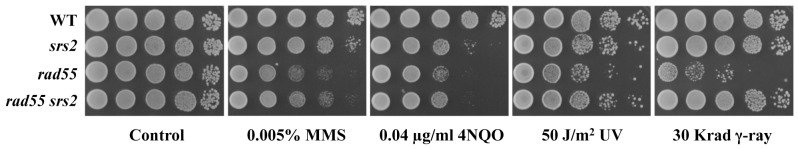
Genetic interactions between *srs2* and *rad55* in response to different types of DNA damage. A serial dilution assay with different treatments shows that *srs2* completely suppresses *rad55* ionizing radiation (IR) sensitivity but barely suppresses the sensitivity to other treatments. Experimental conditions were as described in Figure 1.

In this study, we confirmed a report [24] that *srs2* can suppress the ionizing radiation (IR) sensitivity of the *rad55* mutant (Figure 5). *srs2* also appears to partially suppress *rad55* sensitivity to UV. In contrast, the *srs2 rad55* double mutant is as sensitive to MMS and 4NQO as the *rad55* single mutant (Figure 5), suggesting that this effect is lesion specific.

### Functional Differences between Shu and Rad55–Rad57 Complexes

**Figure 6:**
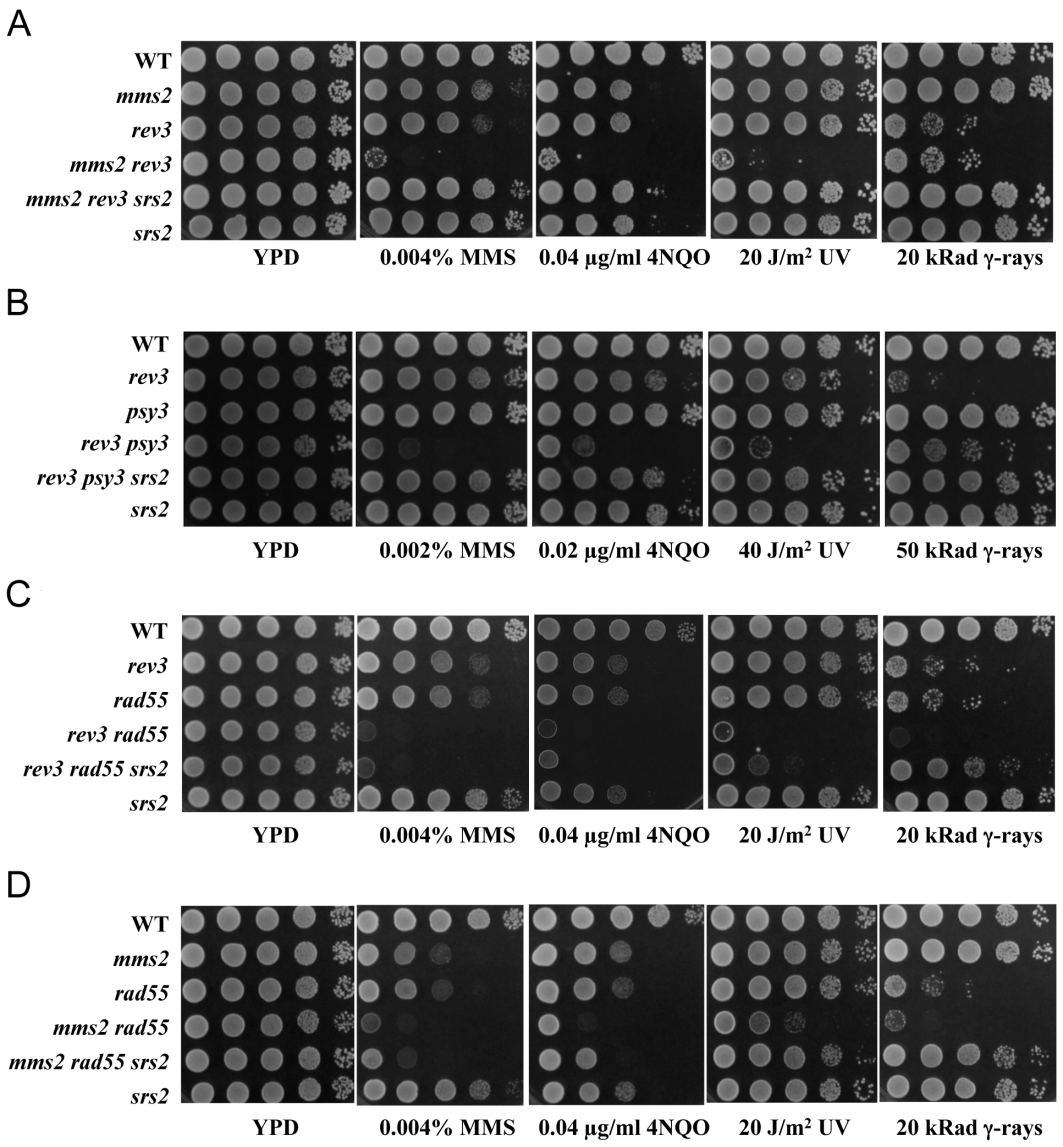
Similarity and differences for the roles of *srs2* mutation in the suppression of various mutants to DNA damage. (A) *mms2* and *rev3*; (B) *psy3* and *rev3*; (C) *rad55* and *rev3*; and (D) *mms2* and *rad55*. Experimental conditions were as described in Figure 1.

Although both physical and genetic studies establish that Shu and Rad55–Rad57 act in the same error-free DDT pathway, phenotypic differences between the two complex mutants are also obvious, which may hold the key to understanding how they are involved in the error-free DDT pathway. To further explore similarities and differences between the two complexes, we sensitized the *mms2/ubc13*, *shu* or *rad55/rad57* single mutants with *rev3* and then examined the ability of *srs2* to suppress such sensitivity. As previously reported [48], the extreme sensitivity of the *mms2 rev3* double mutant is completely suppressed by the *srs2* mutation, regardless of the source of DNA damage (Figure 6A). Similarly, the extreme sensitivity of the *psy3 rev3* mutant is also completely suppressed by the *srs2* mutation under all DNA damage conditions (Figure 6B). In sharp contrast, the extreme sensitivity of the *rad55 rev3* mutant is suppressed by *srs2* only in response to IR-induced DNA damage, but not by other types of DNA damage (Figure 6C).

The *mms2* and *rad55* mutants display comparable levels of sensitivity to MMS, 4NQO and UV irradiation, but the *rad55* mutant is much more sensitive to IR than *mms2* (Figure 6D). In all cases, the two mutations appear to be additive, and the *srs2* mutation suppresses the double mutant to various degrees, ranging from the complete suppression of IR to negligible suppression in MMS (Figure 6D). The above observations collectively indicate that the Rad55–Rad57 complex not only plays a role in error-free DDT, but also in DSB repair and perhaps other pathway(s) independent of DDT.

### The *shu* and *rad55/rad57* Mutations do not Affect DNA Damage-induced PCNA Ubiquitination

**Figure 7:**
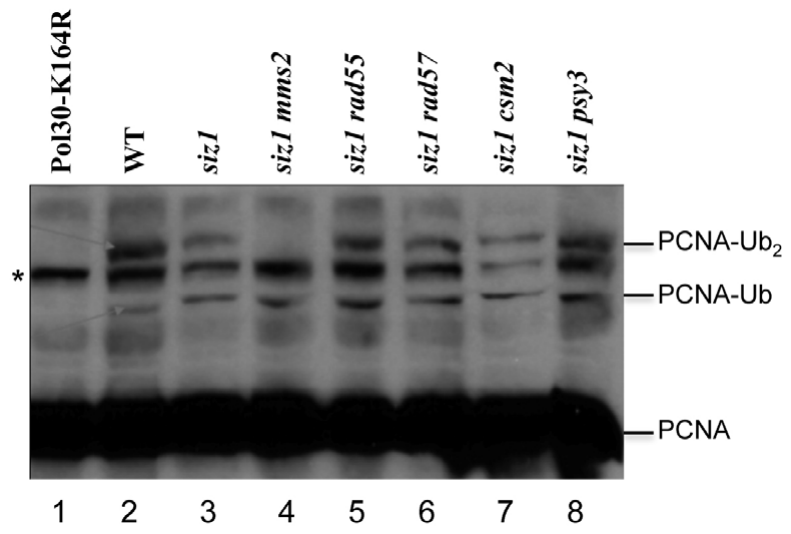
Detection of PCNA ubiquitination by western blotting. Overnight cultures were subcultured until the cell concentration reached approximately 1×10^7^ cells/ml; cultures were then treated with 0.05% MMS for 90 min. Total cell extracts obtained under denaturing conditions were analyzed by western blotting using an in-house-made anti-PCNA monoclonal antibody. Strains used were HKY578-10A (wild-type) and its isogenic derivatives as listed in Supplementary Table S1. PCNA-Ub and PCNA-Ub_2_ refer to the monoubiquitinated and diubiquitinated PCNA, respectively. The *siz1* mutation was used to eliminate endogenous SUMO modification of PCNA, which occurs in both MMS-treated and untreated conditions [5], [9] and co-migrates with diubiquitinated PCNA (data not shown). An asterisk indicates a nonspecific band.

It has been well established that the Rad6–Rad18 complex is required for PCNA monoubiquitination at the K164 residue, and the Mms2-Ubc13-Rad5 complex is required for K63-linked polyubiquitination at the same residue [5]. The *rev1*, *rev3* and *rev7* mutations do not affect PCNA monoubiquitination but abolish TLS. To ask whether Shu and Rad55–Rad57 complexes function upstream or downstream of PCNA polyubiquitination, we examined the effects of individual mutations on the *in vivo* formation of PCNA ubiquitination by a western blotting analysis. To achieve this objective, we first raised and validated a monoclonal antibody against yeast PCNA for its ability to detect endogenous PCNA ubiquitination (Figure S3 in File S1). It was found that under conditions where the Pol30-K164R abolishes both mono- and diubiquitination (Figure 7, lane 1) and the *mms2* mutation only affects PCNA diubiquitination, (lane 4), neither *rad55* (lane 5), *rad57* (lane 6) nor *csm2* (lane 7) or *psy3* (lane 8) affects PCNA ubiquitination. Hence, these two complexes must act downstream of PCNA ubiquitination.

## Discussion

Several observations support a notion that like the Shu complex, the Rad55–Rad57 complex functions in the error-free lesion bypass pathway. Firstly, the observation that *rad55* and *rad57* mutations are epistatic to the *shu* mutations (Figures 1, and Figure S1 in File S1), which are known to function in the error-free DDT pathway, suggests that both function in the same pathway, while the Shu complex has been previously reported to function in the error-free DDT pathway [30]. Secondly, the *rad55* mutation is synergistic to *rev3*, and the synergistic effect is even stronger than that of *mms2/ubc13-rev3* and *shu-rev3* (Figure 3). This is a characteristic feature of error-free DDT pathway mutations [30], [35], [37]. Thirdly, the *rad55* and *rad57* mutants also display a *REV3*-dependent increase in spontaneous mutagenesis (Figure 2), another characteristic feature of error-free DDT mutants [30], [35]. Finally, the physical interaction between Csm2 and Rad55 (Figure 4) bridges the above two complexes into one mega-complex and provides a mechanistic link from error-free DDT to HR.

**Figure 8:**
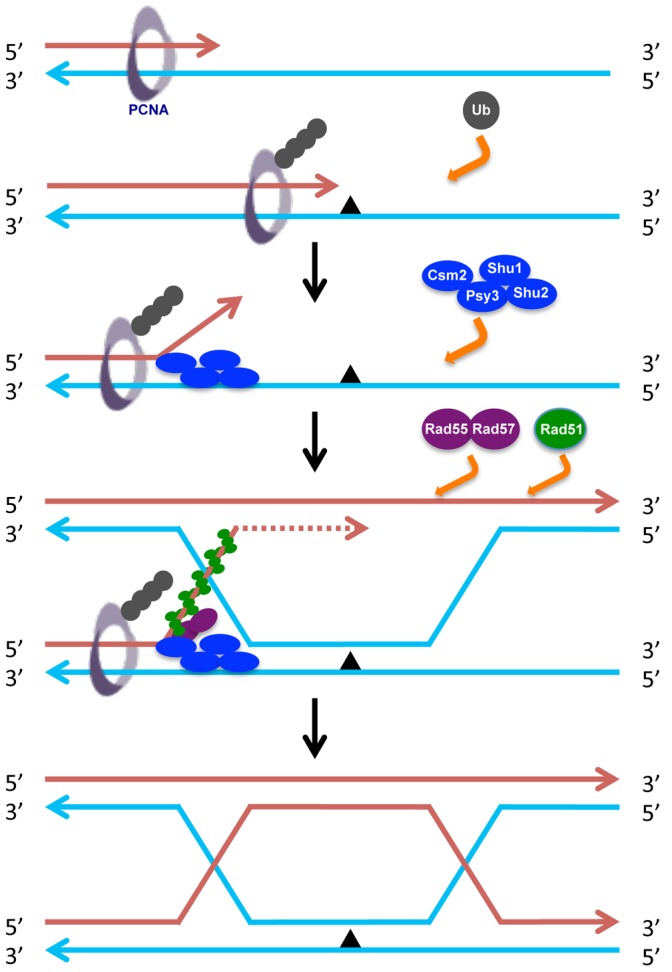
A working model illustrating the coordinated activities of Shu and Rad55–Rad57 complexes in the error-free bypass of replication-blocking lesions. When a replication fork encounters a replication block, PCNA can be polyubiquitinated, which recruits the Shu complex and in turn recruits Rad55–Rad57 through the Csm2-Rad55 physical interaction. Both complexes facilitate Rad51-ssDNA filament formation and promote template switching for error-free lesion bypass. Note that this diagram does not illustrate how the two complexes antagonize Srs2 activity. Also not illustrated is that an alternative solution to the stalled replication fork is through PCNA monoubiquitination and TLS.

Unlike the *mms2*, *ubc13* or *rad5* mutations, the *rad55/rad57* or *shu* mutations do not affect MMS-induced PCNA polyubiquitination at the K164 residue (Figure 7), indicating that the two complexes function downstream of PCNA polyubiquitination, reminiscent of the relationship between TLS and PCNA monoubiquitination. The two complexes must act together to couple error-free DDT to HR for the bypass of replication-blocking lesions. Based on the phenotypic differences between *shu* and *rad55/rad57* mutants, we further argue that the Shu complex acts upstream of the Rad55–Rad57 complex in this process. Firstly, the *rad55/rad57* mutant is more sensitive to DNA damage than the *shu* mutant and *rad55/rad57* is completely epistatic to *shu* (Figures 1, and Figure S1 in File S1), suggesting that Shu is an accessory factor for the Rad55–Rad57 complex. Secondly, the *shu* mutant behaves more like the *mms2/ubc13* mutant than the *rad55/rad57* mutant, while the *rad55/rad57* mutant is more like the *hr* mutant than the *shu* mutant. Most striking is that unlike the *rad55/rad57* mutant, neither *mms2* nor *shu* mutant displays noticeable sensitivity to γ-rays under our experimental conditions (Figure 6). Finally, although both *mms2* [48] and *shu* [30] sensitivities were completely suppressed by the *srs2* mutation (Figure 6), the [42] *srs2* mutation only completely suppressed the γ-ray sensitivity of *rad55*, but not sensitivity to other types of DNA damage (Figure 5). Since DNA damage induced by γ-rays is mainly repaired by HR while damage induced by UV, 4NQO and MMS is largely considered replication blocks, the above observations further reinforce the notion that Shu and Rad55–Rad57 work together at the stalled replication site to recruit HR for an error-free lesion bypass using newly synthesized sister chromatid as a template. Such a model is illustrated in Figure 8, in which the Shu complex is thought to be recruited to the forked or 3′ overhang DNA [42], where it facilitates Rad51-ssDNA filament formation. This may be achieved by displacing RPA [23], antagonizing Srs2 [27], recruiting the Rad55–Rad57 complex [42] and/or stabilizing the Rad51-ssDNA filament formation [49].

Both Shu [27] and Rad55–Rad57 [24] complexes have been implicated in the inhibition of Srs2 activity. The above model does not elucidate how the inhibition is achieved. Our observations indicate that the *srs2* mutation effectively suppresses all *shu* mutants regardless of the type of DNA damage (Figure 6). On the other hand, there are at least two modes of genetic interactions between *rad55/rad57* and *srs2*. For IR-induced DNA damage, *srs2* completely suppresses *rad55/rad57* phenotypes, confirming the anti-Srs2 activity of Rad55–Rad57 [24]. For MMS- or 4NQO-induced DNA damage, *srs2* does not suppress the *rad55* mutant phenotypes in a wild type or *rev3* background (Figure 5). We argue that the above different modes of genetic interaction are due to dealing with different type of DNA lesions, namely DSB *vs*. stalled replication.

While this research was in progress, Bernstein and colleagues reported the physical interaction between Csm2 and Rad55, and the epistatic relationship of the two complex mutations [42]. While our results support some of the above report, our interpretation based on additional observations is that the interactions between the two Rad51 paralogues mainly deal with stalled replication forks instead of DSBs. In particular, the striking synergistic interaction between *rad55/rad57* and *rev3* in terms of DNA damage response, and the Rev3-dependent increase in spontaneous mutagenesis have to be explained. Since TLS does not readily bypass DSBs, the explanation we favor is a common substrate such as a stalled replication fork in front of a replication lesion (Figure 8).

## Materials and Methods

### Media and Strains

The yeast strains used in this study are listed in Table S1 of File S1. All the strains used were generated from DBY747, HK578 or BY4741 backgrounds. All of the BY4741 single mutant derivatives were created by the Saccharomyces Genome Deletion Project Consortium and purchased from Research Genetics (Invitrogen, Carlsbad, CA, USA). Other gene deletion mutants were created by a single-step gene disruption method [50] using synthetic oligonucleotides or disruption cassettes designed in-house, or combined through crossing isogenic single mutant strains. All the gene deletion strains were confirmed by genomic PCR prior to functional characterization.

Yeast extract/peptone/dextrose (YPD) medium and SD medium supplemented with required amino acids were used to culture yeast cells [51]. Yeast transformation was carried out following a modified lithium acetate method [52].

### Yeast Two-hybrid Analysis

Plasmids containing *SHU* genes for the yeast two-hybrid (Y2H) assay are as previously described [30]. All the other Y2H plasmids were constructed by cloning ORFs of each gene into pGBT9 (Gal4_BD_) or pGAD424 (Gal4_AD_). Y2H plasmids were transformed into PJ69-4a [53] in pairs and allowed to grow at 30°C for 2–3 days. Transformants were scored for growth on selective media, followed by phenotypic characterization.

### Coimmunoprecipitation

The 3xFlag-His_6_-EGFP sequence from plasmid pGFP(S65T)-3F6H/TADH1/URA3 [54] was integrated at 3′ end of the chromosomal *CSM2* ORF in the yeast strain BY4741 to express a Csm2-yEGFP protein. Plasmid pGAD-Rad55 or an empty vector pGAD424 was transformed into the resulting strain (Table S1 in File S1). Confirmed transformants were cultured overnight at 30°C followed by subculture until logarithmic phase, when total proteins were extracted in a lysis buffer (Novagen YeastBuster Protein Extraction Reagent, Cat. 71186) and incubated with GFP-Trap®A (gta-20, ChromoTek) overnight. Beads were boiled with a 5x loading buffer for 5 min and then washed three times with the lysis buffer. The input and the coimmunoprecipitated proteins were detected by western blotting with an anti-Gal4_AD_ antibody (630402, Clontech).

### Testing the Sensitivity to DNA-damaging Treatments

The gradient plate assay and serial dilution assays were carried out as previously described [55]. The MMS-induced liquid killing assay followed the protocol previously used [56]. Cells were inoculated into YPD and cultured at 30°C overnight. A predetermined volume of MMS was added to the liquid culture and incubation continued until cells were washed and harvested by centrifugation, followed by plating on YPD. The number of colonies was counted after a 3-day incubation.

### Spontaneous Mutagenesis Assay

The spontaneous mutagenesis rate was reflected by the reverse mutation rate of the *trp1*-289 allele in the DBY747 strain. This method is a modified Luria and Delbruck fluctuation test, as previously described [35].

### Detection of PCNA Ubiquitination

The method used to detect PCNA ubiquitination in this study was adapted from a previous report [57]. Cells were cultured in YPAD (YPD +20 mg/mL Ade) overnight and then diluted to 0.3 x 10^7^ cells/mL in 100 mL YPAD and continue incubating in 30°C for 2 hours. Cultures were then treated with 0.05% MMS for 90 minutes. 20 mL of the culture was harvested by centrifugation and immediately frozen in liquid nitrogen for 10 minutes. After resuspension in N-ethylmaleimide (NEM) plus phenylmethylsulfonyl fluoride (PMSF), the cells were lysed with NaOH plus 7.5% β-mercaptoethanol, and then precipitated with trichloracetic acid. The pellet was resuspended in HU buffer (8 M Urea, 5% SDS, 200 mM Tris-HCL pH 6.8, 1 mM EDTA, 0.025% bromophenol blue, 1.5% DTT, 25 mM NEM, 1 mM PMSF, and 0.5% triton-X-100) and then denatured by heating. Laemmli sample buffer (Bio-Rad) was added to the sample, which was then frozen overnight before SDS-PAGE and western blotting. In-house-made Pol30 monoclonal antibodies were used in the western-blot assay.

## Supporting Information

File S1 **Figure S1 Epistasic relationship between** ***shu*** **and** ***rad55/57*** **, as shown by a gradient plate assay. Figure S2 Genetic relationships between** ***mms2*** **,** ***rev3*** **and HR gene mutations. Figure S3 Control experimental data to confirm anti-PCNA antibody and detection of PCNA ubiquitination. Table S1** ***S. cerevisiae*** **strains.**
(DOCX)
