# Supplementary material for: The Yeast Shu Complex Utilizes Homologous Recombination Machinery for Error-free Lesion Bypass via Physical Interaction with a Rad51 Paralogue
Source: PLoS One. 2013 Dec 5;8(12):e81371. doi: 10.1371/journal.pone.0081371 (PMC3855272; doi:10.1371/journal.pone.0081371)
Supplement: File S1 — Figure S1 Epistasic relationship between shu and rad55/57 , as shown by a gradient plate assay. Figure S2 Genetic relationships between mms2 , rev3 and HR gene mutations. Figure S3 Control experimental data to confirm anti-PCNA antibody and detection of PCNA ubiquitination. Table S1 S. cerevisiae strains. (DOCX) [file pone.0081371.s001.docx]

**Supporting Information**

**Supporting Information Legends**

**Figure S1.** Epistasic relationship between *shu* and *rad55/57*, as shown by a gradient plate assay. Overnight cell cultures were imprinted on YPD or YPD + MMS and incubated at 30°C for 2 days before photography. Arrows indicate the increasing concentration of MMS.

**Figure S2**. Genetic relationships between *mms2*, *rev3* and HR gene mutations. Experimental conditions were as described in Figure S1.

**Figure S3**. Control experimental data to confirm anti-PCNA antibody and detection of PCNA ubiquitination. Overnight cultures were subcultured and allowed to grow to a cell count of approximately 1x10^7^cells/ml before being treated with 0.05% MMS (as indicated) for 90 minutes. Total cell extracts were obtained under denaturing conditions and analyzed by SDS-PAGE and western blot. Yeast strains and treatment conditions are as indicated on the top panel.


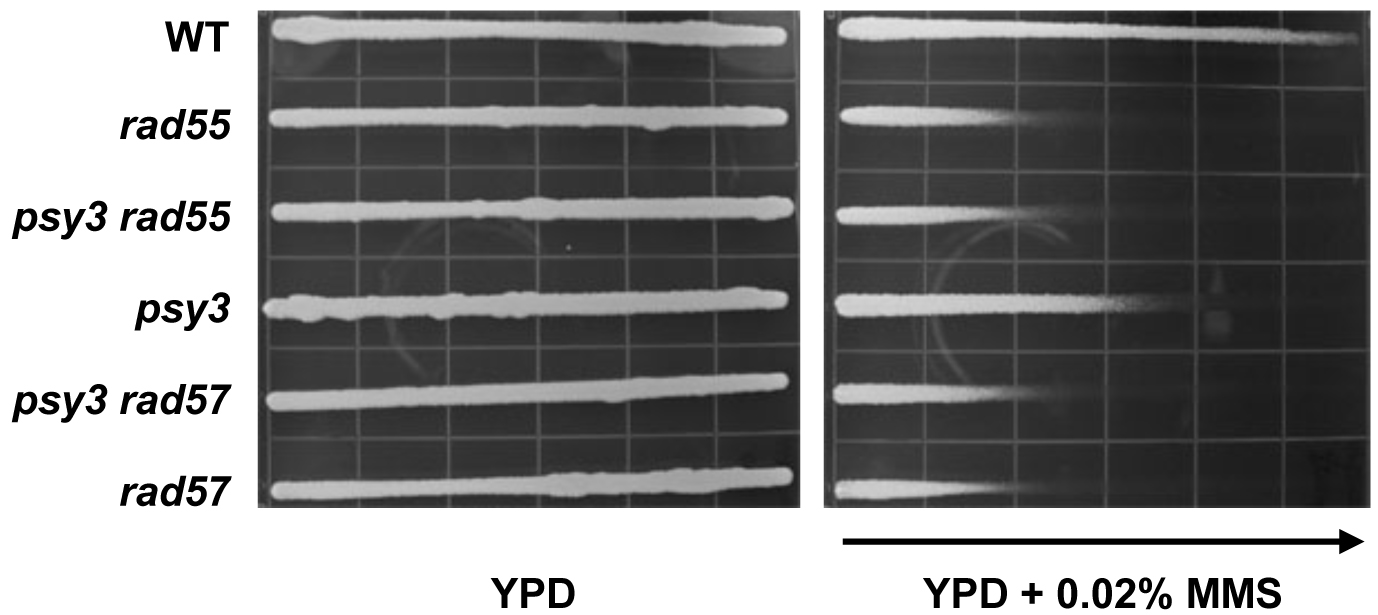


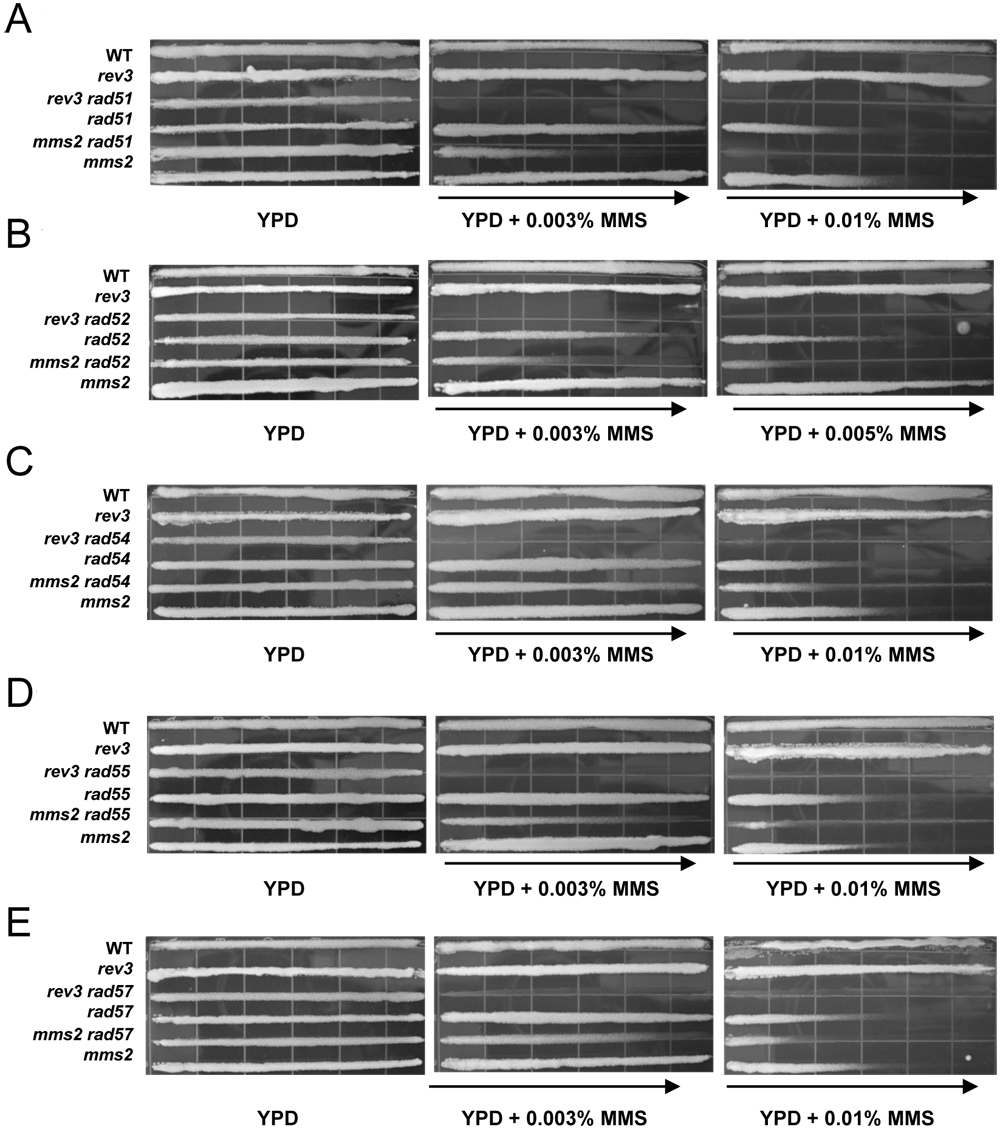


Figure S1


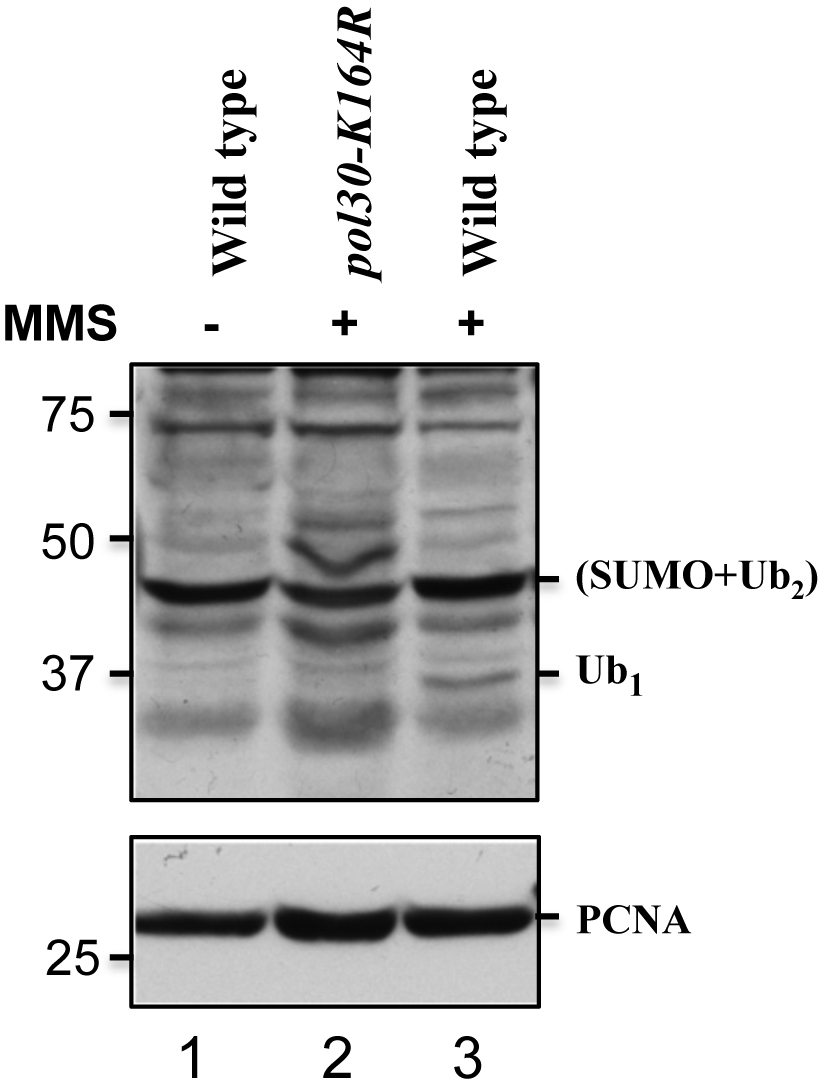


Figure S2

Figure S3

**Table S1.** *S. cerevisiae* strains

| **Strain** | **Genotype** | **Source** |
| --- | --- | --- |
| BY4741 | *MAT***a** *his3Δ1 leu2Δ0 met15Δ0 ura3Δ0* | SGD Consortium |
| BY4741 *trp1Δ* | BY4741 with *trp1Δ::Kan^R^* | SGD Consortium |
| BY4741 *psy3Δ* | BY4741 with *psy3Δ::Kan^R^* | SGD Consortium |
| BY4741 *rad51Δ* | BY4741 with *rad51Δ::Kan^R^* | SGD Consortium |
| BY4741 *rad52Δ* | BY4741 with *rad52Δ::Kan^R^* | SGD Consortium |
| BY4741 *rad54Δ* | BY4741 with *rad54Δ::Kan^R^* | SGD Consortium |
| BY4741 *rad55Δ* | BY4741 with *rad55Δ::Kan^R^* | SGD Consortium |
| BY4741 *rad57Δ* | BY4741 with *rad57Δ::Kan^R^* | SGD Consortium |
| BY4741 *mms2Δ* | BY4741 with *mms2Δ::Kan^R^* | SGD Consortium |
| BY4741 *rev3Δ* | BY4741 with *rev3Δ::Kan^R^* | SGD Consortium |
| BY4741 *srs2Δ* | BY4741 with *srs2Δ::Kan^R^* | SGD Consortium |
| HKY579-10A | *MATa ade2-1 can1-100 his3-11,15 leu2-3,112 trp1-1 ura3-1* | H. Klein |
| WXY2960 | HKY579-10A with *siz1∆::HIS3 mms2∆::URA3* | This study |
| WXY3069 | HKY579-10A with *siz1Δ::LEU2 rad55Δ::HIS3* | This study |
| WXY3068 | HKY579-10A with *siz1Δ::HIS3 rad57Δ::LEU2* | This study |
| WXY2970 | HKY579-10A with *siz1∆::HIS3 csm2∆::LEU2* | This study |
| WXY2967 | HKY579-10A with *siz1∆::HIS3 psy3∆::URA3* | This study |
| WXY3067 | HKY579-10A with *siz1Δ::LEU2* | This study |
| WXY994 | HKY579-10A with *pol30-K164R* | Lab stock |
| Deletion mutant array (DMA) | BY4741 *can1∆::MFA1pr-HIS3 lrp1∆* | Lab stock |
| WXY2469 | BY4741 with *mms2*∆::*Kan^R^ rad51*∆::*LEU2* | This study |
| WXY2416 | DMA with *mms2∆::Nat^R^ rad52∆::Kan^R^* | This study |
| WXY2417 | DMA with *mms2∆::Nat^R^ rad54∆::Kan^R^* | This study |
| WXY2419 | DMA with *mms2∆::Nat^R^ rad55∆::Kan^R^* | This study |
| WXY2420 | DMA with *mms2∆::Nat^R^ rad57∆::Kan^R^* | This study |
| WXY3109 | BY4741 with *rad55∆::Kan^R^ rev3∆::Nat^R^ srs2∆::LEU2* | This study |
| WXY3092 | BY4741 with *rad55∆::Kan^R^ srs2∆::Nat^R^* | This study |
| WXY2539 | BY4741 with *mms2∆::Kan^R^ rev3∆::LEU2* | This study |
| WXY3108 | BY4741 with *rev3∆::Kan^R^ mms2∆::URA3 srs2∆::LEU2* | This study |
| WXY2219 | DMA with *rev3∆::Nat^R^ rad51∆::Kan^R^* | This study |
| WXY2032 | DMA with *rev3∆::Nat^R^ rad52∆::Kan^R^* | This study |
| WXY2555 | DMA with *rev3*∆::*Nat^R^ rad54∆::Kan^R^* | This study |
| WXY2020 | DMA with *rev3*∆::*Nat^R^ rad55∆::Kan^R^* | This study |
| WXY2421 | DMA with *rev3*∆::*Nat^R^ rad57∆::Kan^R^* | This study |
| WXY3005 | BY4741 with *rad55∆::Kan^R^ psy3∆::HIS3* | This study |
| WXY3006 | BY4741 with *rad57∆::Kan^R^ psy3∆::HIS3* | This study |
| WXY3114 | BY4741 with *rev3∆::Kan^R^ mms2∆::URA3 psy3∆::HIS3* | This study |
| WXY1333 | BY4741with *rev3∆::Kan^R^ psy3∆::HIS3* | Lab stock |
| WXY3111 | BY4741 with *mms2∆::Kan^R^ srs2∆::LEU2 rad55∆::HIS3* | This study |
| WXY3515 | BY4741 with *CSM2::EGFP-URA3* | This study |
| PJ69-4a | *MAT***a** *trp1-901 leu2-3,112 ura3-52 his3-200 gal4Δ gal80Δ Met2::GAL7-lacZ LYS2::GAL1-HIS3 GAL2-ADE2* | P. James |
| PJ69-4a *rad55Δ* | PJ69-4a with *rad55Δ:: Nat^R^* | This study |
| PJ69-4a *rad51Δ* | PJ69-4a with *rad51Δ:: Nat^R^* | This study |
| DBY747 | *MAT***a** *his3-Δ1 leu2-3,112 trp1-289 ura3-52* | P. James |
| WXY1323 | DBY747 with *psy3Δ::Kan^R^* | Lab stock |
| WXY667 | DBY747 with *rev3Δ::hisG-URA3-hisG* | Lab stock |
| WXY644 | DBY747 with *mms2Δ::URA3* | D. Botstein |
| WXY3053 | DBY747 with *rad55Δ::HIS3* | This study |
| WXY3055 | DBY747 with *psy3Δ::Kan^R^ rad55Δ::HIS3* | This study |
| WXY3059 | DBY747 with *rad55Δ::HIS3 rev3Δ::LEU2* | This study |
| WXY3054 | DBY747 with *rad57Δ::LEU2* | This study |
| WXY1164 | DBY747 with *rad51Δ::HIS3* | This study |
| WXY3057 | DBY747 with *psy3Δ::Kan^R^ rad57Δ::LEU2* | This study |
| WXY3061 | DBY747 with *mms2∆::URA3 rad55∆::HIS3* | This study |
| WXY3060 | DBY747 with *rad57Δ::LEU2 rev3Δ::hisG-URA3-hisG* | This study |
| WXY3062 | DBY747 with *mms2∆::URA3 rad57∆::LEU2* | This study |
